# An expert assessment on climate change and health – with a European focus on lungs and allergies

**DOI:** 10.1186/1476-069X-11-S1-S4

**Published:** 2012-06-28

**Authors:** Bertil Forsberg, Lennart Bråbäck, Hans Keune, Mike Kobernus, Martin Krayer von Krauss, Aileen Yang, Alena Bartonova

**Affiliations:** 1 Occupational and Environmental Medicine, Umea University, Sweden; 2 Research Institute for Nature and Forest (INBO), Brussels; Centre of Expertise for Environment and Health, Faculty of Political and Social Sciences, University of Antwerp; naXys, Namur Center for Complex Systems, University of Namur, Belgium; 3 NILU – Norwegian Institute for Air Research, Kjeller, Norway; 4 WHO Euro, Copenhagen, Denmark

## Abstract

**Background:**

For almost 20 years, the Intergovernmental Panel on Climate Change has been assessing the potential health risks associated with climate change; with increasingly convincing evidence that climate change presents existing impacts on human health. In industrialized countries climate change may further affect public health and in particular respiratory health, through existing health stressors, including, anticipated increased number of deaths and acute morbidity due to heat waves; increased frequency of cardiopulmonary events due to higher concentrations of air pollutants; and altered spatial and temporal distribution of allergens and some infectious disease vectors. Additionally exposure to moulds and contaminants from water damaged buildings may increase.

**Methods:**

We undertook an expert elicitation amongst European researchers engaged in environmental medicine or respiratory health. All experts were actively publishing researchers on lung disease and air pollution, climate and health or a closely related research. We conducted an online questionnaire on proposed causal diagrams and determined levels of confidence that climate change will have an impact on a series of stressors. In a workshop following the online questionnaire, half of the experts further discussed the results and reasons for differences in assessments of the state of knowledge on exposures and health effects.

**Results:**

Out of 16 experts, 100% expressed high to very high confidence that climate change would increase the frequency of heat waves. At least half expressed high or very high confidence that climate change would increase levels of pollen (50%), particulate matter (PM2.5) (55%), and ozone (70%). While clarity is needed around the impacts of increased exposures to health impacts of some stressors, including ozone and particulate matter levels, it was noted that definitive knowledge is not a prerequisite for policy action. Information to the public, preventive measures, monitoring and warning systems were among the most commonly mentioned preventative actions.

**Conclusions:**

This group of experts identifies clear health risks associated with climate change, and express opinions about these risks even while they do not necessarily regard themselves as covering all areas of expertise. Since some changes in exposure have already been observed, the consensus is that there is already a scientific basis for preventative action, and that the associated adaptation and mitigation policies should also be evidence based.

## Background

For almost 20 years, the Intergovernmental Panel on Climate Change (IPCC) has been assessing the potential health impacts of climate change, with increasingly convincing evidence that climate change presents existing risks to human health and that without timely and effective interventions, these risks will increase with additional climate change [1].

According to the summary statements from the Intergovernmental Panel on Climate Change 4th Assessment Report [1]: over the past 50 years, it is very likely (defined as >90% likelihood) that hot days and hot nights became more frequent, and it is likely (>66% likelihood) that heat waves will become more frequent over most land areas. It is very likely that heavy precipitation events will become more frequent; and likely that tropical cyclones will become more intense, with larger peak wind speeds and heavier rainfall; that in areas already affected by drought will increase; as will the incidence of coastal flooding from extremely high sea levels.

However, the changes in climate will differ by region. The increase in temperature will be greater at higher latitudes. The estimated increases in extreme precipitation are much larger for northern Europe than in southern Europe [2]. Modeled estimates of climate change induced increases in near-surface ozone concentrations and accumulated ozone, exposure over a threshold of 40 ppb (ppb hrs), are much larger in southern Europe [3].

Warnings from experts on health threats have become increasingly dire. McMichael et al in 2006 stressed that climate change will affect human health in many ways [4]. In this paper the authors discussed the problems of detecting global warming effects on health outcomes at an early stage, but showed that estimations in some cases are possible. They also concluded that research on climate change and health risk so far has mostly focused on thermal stress, extreme weather events, and infectious diseases and are lacking in other areas.

Given the observed and predicted detrimental health impacts of climate change, broadening the current focus within the public climate discourse is a an important challenge for the health sector [4,5]. Although most of the adverse effects of climate change will threaten human health, the assessments that have gained most attention from governments have focused mainly on economic effects, suggesting that the economy was the most important issue for society. Experts in environmental health and public health agencies need to engage further in the process of understanding and communicating the implications of climate change on public health and wellbeing. Recently there have been an increasing number of initiatives by health scientists and physicians designed to increase the public interest of the threat.

A recent position statement on climate change and health impacts from the European Respiratory Society (ERS) was developed after a workshop co-organized by the HENVINET Project and the American Thoracic Society [6]. The position statement highlights climate related health impacts, including deaths and acute morbidity due to heat waves; increased frequency of acute cardio-respiratory events due to higher concentrations of ground level ozone; changes in the frequency of respiratory diseases due to transboundary particle pollution; and altered spatial and temporal distribution of allergens (pollens, mold and mites) and some infectious disease vectors. According to the report these impacts will not only affect those with existing respiratory disease but will likely increase the incidence and prevalence of respiratory conditions.

The effect of heat waves on mortality is well documented [7]. The increase in respiratory mortality (relative risk) is larger than total or cardiovascular mortality [8]. Although the association between heat and the number of hospital admissions is less studied, and less evident, admissions are, however, also more apparent for respiratory disease than for cardiovascular [9].Air pollution is the environmental factor with the greatest impact on respiratory health in Europe. Particle pollution, vehicle exhaust and ground level ozone are the most important types of hazardous pollutants. Pollution models for climate change scenarios predict an increase in ozone concentrations over large areas, while the effect on particle concentrations is less clear [10]. Higher temperatures, clear skies and stagnant conditions will favor ozone production. The short-term effects of ozone on daily mortality [11] and respiratory disease [12] are extensively studied, while there is only limited documentation of long-term effects on mortality [13].

The climate in general and weather extremes may have an effect on allergic diseases and asthma via the impact on allergen exposures. Higher temperatures and concentrations of CO_2_ are associated with an increase in pollen production [14], and with climate change the timing of the pollen season may change [15]. Heavy rain and flooding may cause water damage on buildings and lead to increased mould exposure. Although mould allergy is rare there is a clear relationship between damp houses and respiratory diseases such as asthma [16]. Additionally while asthma in children and young adults has been less common in areas with colder winters and lower humidity than along the wetter coastal areas [17,18], more severe rainfall and storms could increase this risk. House dust mites are rare in cold winter climates in the north and at high elevation where the heated indoor air becomes dry in winter. A cold winter could be enough to reduce exposure to mite allergens [17], however, with milder winters mite allergies may become worse and more common.

Since mortality is higher in the cold season, without also considering influenza epidemics, and cold spells associated with greater mortality, a milder winter could result in less cold-related mortality especially in countries not well adapted to cold [19].

Despite the likelihood that most of the adverse effects of climate change will threaten human health, health effects have not featured greatly in the climate discourse. Therefore we wanted to study how health experts look upon the health risks, and upon knowledge gaps. We also wanted to identify potential differences of opinion amongst scientists. Since there is a large body of literature on air pollution levels, allergens and respiratory morbidity and mortality and potential health effects of climate change, we sought the opinions of experts in these fields for our study.

## Methods

We used an expert elicitation method for assessment of knowledge on climate change and health risks [20]. On the basis of the literature on climate change and health risks in Europe, a causal diagram (proposed pathways to health effects) outlining eight different pathways to asthma/allergies and other respiratory endpoints was developed. The causal diagram was presented in an online questionnaire, accompanied by general motivations without a presentation of supporting references [21]. The causal pathways dealt with extreme heat, extreme cold, ozone, particulate pollution, allergenic pollens, mould spores, damp buildings and dust mites. A first test of the online questionnaire was organized in 2008 among a group of participants registered for the European Respiratory Society workshop [6]. The questions were formulated based on the rating of confidence levels inspired by the IPCC quantitatively calibrated levels of confidence [22]. Each relationship in the causal model had a corresponding question, for example: “What is your level of confidence in the claim that increased levels of secondary fine particles also will result in an increased population exposure?” The respondent’s confidence in current scientific methods for predicting the magnitude of the effect could be assessed as very high (at least a 9 out of 10 chance of being correct), high (at least an 8 out of 10), medium (at least a 5 out of 10), low (at least a 2 out of 10) or very low (less than a 1 out of 10). In the analysis we coded the score “very high” = 5, “high” = 4 and so on down to “very low” = 1. We analysed the consensus for answers in the online questionnaire using a consensus index following the method proposed by Tastle and Wierman [23]. This index attains consensus values between 0 (perfect disagreement) and 1 (perfect agreement). The test among workshop participants resulted only in minor revisions in the formulating of questions/claims, in particular to make it clear that potential changes in exposure or health due to other reasons than climate change were not included in the claims, and that the individual sets of questions were to be treated independently irrespective of the state of knowledge of other elements of the diagram.

The online questionnaire also asked to rank from 1 (highest importance) to 8 (least importance) the relative importance of the health impact to be expected via each pathway in comparison with the others. The questionnaire moreover asked: “Does the diagram take into account all the important parameters…” where the answer “no” was followed by a request for comments. Another question on the causal model was: “Are the different causal relationships adequately structured? If no please explain!”

For the 2009 study we invited 48 experts in the field of respiratory and environmental medicine, public health and/or epidemiology. All invited experts had recent publications listed in PubMed on asthma and air pollution or climate change, and had been studying European populations. They could all be considered health experts with expertise relevant for an assessment of potential health impacts related to climate change in their country or Europe in general.

**Figure 1:**
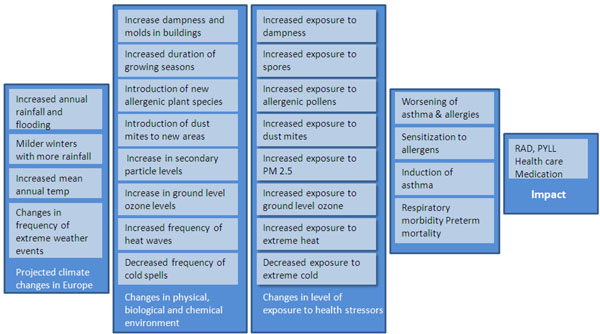
**Overall causal model** Overall causal model for how climate change, stressor level, population exposure, morbidity and other health impacts are linked. The figure shows the claims and questions. The detailed 8 pathways, claims and related questions are not shown in the figure, see reference [21].

Sixteen out of 48 experts accepted the invitation to participate in the online evaluation of the revised causal diagram with proposed relationships and the associated questionnaire (Figure 1). The participating experts are listed in appendix 1. Nine of the 16 experts also responded to a second questionnaire on the kind of policy action they considered justifiable based on the identified state of scientific knowledge, thereby determining the applicability of the current evidence to health policy [20]. In a follow-up workshop held two months later in September 2009, eight of these nine experts discussed the outcomes of the first and the second questionnaire. The workshop was organized parallel to the annual conference arranged by the European Respiratory Society, with a focus on respiratory health.

## Results

### Knowledge evaluation

#### Ability to predict the magnitude of changes

**Figure 2:**
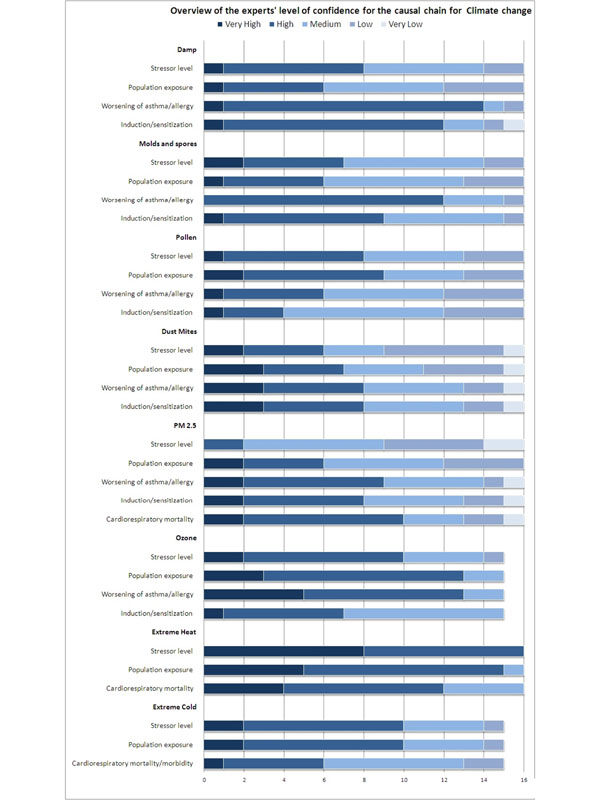
**Evaluation of the proposed relationships in the causal diagram on asthma and allergies** Evaluation of the proposed relationships in the causal diagram used this study by 16 experts.

As shown in Figure 2, the participating scientists rated with high confidence the ability of current scientific methods to predict the magnitude of the change in the frequency and duration of heat waves (mean score 4.5), and increase in population exposure to extreme heat (mean score 4.25). The mean score was also high for the ability to predict the magnitude of the increase in the frequency of acute asthma and respiratory morbidity as a result of increased exposure to ozone (mean score 4.2), and for the previous link in the pathway, the increase in exposure to ozone (mean score 4.07). Mean scores of 4 or higher were found for the two causal pathways related to heat and ozone, indicating high confidence. Levels of particles, as PM_2.5_, (mean score 2.56) and the distribution and levels of house dust mites rated a mean score of 3.0 indicating moderate confidence. The latter two pathways were overall considered to be poorly understood due to lack of evidence from relevant studies.

#### Consensus in judgements

The consensus was highest for the ability of current scientific methods to predict the magnitude of the increase in the frequency of acute asthma and respiratory morbidity as a result of increased exposure to mould and spores in buildings (Figure 2), where 13 out of 16 experts answered that we have high ability to predict the magnitude of this effect (consensus index 0.85). Second highest consensus was seen for confidence of scientific methods of predicting the magnitude of the increase in population exposure to ground-level ozone, where 10 experts answered high ability, three answered very high and two medium high (consensus index 0.85). For questions dealing with house dust mites and PM_2.5_ the consensus among experts was generally the lowest in this study.

#### Relative importance of stressors

When the respondents in the questionnaire had to rank (from one (highest) to eight (lowest)) the relative importance of the health impacts of the various pathways, extreme heat stood out as most important, with 3.25 as the mean rank, and the first rank by 7 out of 16 experts. Thereafter followed ozone (3.94), PM_2.5_ (4.19) and ranked most important by three experts, damp buildings (4.63), pollen (4.69), mould and spores (4.88), extreme cold (5.38) and dust mites (5.69). Extreme heat, extreme cold, PM2.5, mould and spores and, damp buildings had all been ranked both as the most and least important climate related pathway to health impacts.

Among the experts that later participated at the follow-up workshop the highest ranking was given to extreme heat (2.89), ozone (4.33), damp buildings (4.33) and PM2.5 (4.56).

#### Comments on the causal diagram

Eight out of sixteen respondents considered that all the important parameters were taken into account, while the other half had additional comments. Their recommendations included: broadening the focus from asthma and allergy; considering the effects of drought, thunderstorms, psychosocial stress, other air pollutants (coarse particles and emissions from heating), infectious agents and adaptation (air conditioning); as well as variations in susceptibility, and the potential positive effects on respiratory infections and allergies. Two respondents additionally recommended building a more complex model with a network of arrows or feed- back loops.

### Policy interpretation questionnaire

#### Most important causal elements

The experts participating in answering the second questionnaire very clearly considered that “exposure” to be the most important element to the influence health within the causal diagram. For several specific exposure elements the following specific statements were made. With respect to ozone, there is sufficient evidence for the causal diagram on health outcomes. Furthermore relatively small changes will induce changes in health outcomes. Moreover, the ozone impact will increase with rising temperatures. There is however a need for research to clarify seasonal variations in ozone, the influence of sunshine and chemicals, and long term effects of ozone. With respect to dampness, there is enough evidence for health effects, as small changes in exposure will have effects on asthma. In Europe the risk of flooding is generally considered to be lower than the risk of drought, thus limiting exposure to a fraction of population, which may lower the priority of flood related health policy action. Regarding extreme heat, health impacts particularity in the elderly population are expected increase, and there is sufficient evidence for respiratory and other health effects. With respect to PM 2.5 the consensus was that there is sufficient evidence that small changes will induce changes in many health outcomes for the general population. While there is sufficient evidence that pollen exposures are expected to increase with climate change, and that this will impact on the large population of people with pollen allergy, the health impact is considered to be a limited issue due to its seasonal nature.

#### Policy action

A wide range of policy actions is covered by the response of the experts, ranging from fundamental and applied scientific research to concrete policy actions, both monitoring and awareness raising, and restrictive or prohibiting activities. We discuss a few concrete examples. Regarding ozone, extreme heat and pollens specifically, a combination of monitoring and warning systems and medical advice is proposed. Regarding ozone, specifically the problem of conflicting data is mentioned as a problem to be solved. Better insulation against heat is specifically mentioned regarding extreme heat events. With respect to dampness a wide range of actions is mentioned: indoor ventilation, water leak repairs, insulation, better heating, implementing best practices, better standardized detection, prohibiting risk activities indoor and outdoor, awareness raising and testing buildings for extreme weather conditions. PM_2.5_ is considered best handled by the following types of actions: congestion pricing, clean cars, less power plant emissions and prohibiting risk activities indoor and outdoor.

#### Confidence in science and policy; weight of knowledge

**Figure 3:**
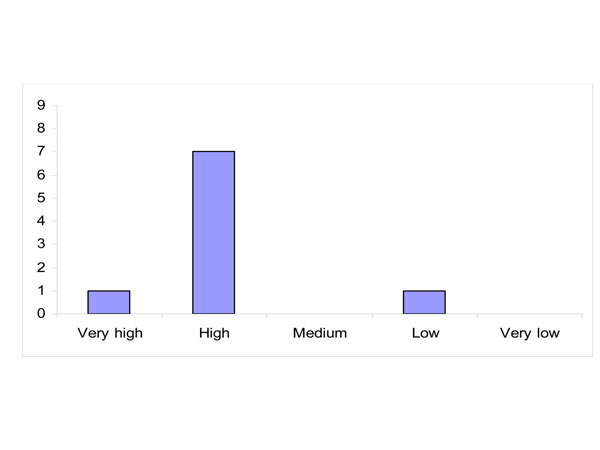
Level of confidence whether conducting more scientific research will yield decisive knowledge within the next five years (distribution of answers)

Most participating experts have high confidence that conducting more scientific research will yield decisive knowledge within the next five years (Figure 3).

Reasons given for a rating of high confidence was that although a five year period is short it is enough time for research to produce results. Furthermore the available mechanistic knowledge (or confidence in causal pathways) is considered a basis for preventive actions, and the available evidence is considered sufficient for policy action, even if there is still a need for “action knowledge” to be further researched. One expert expressed low confidence with the concern that policy is rarely evidence based.

**Figure 4:**
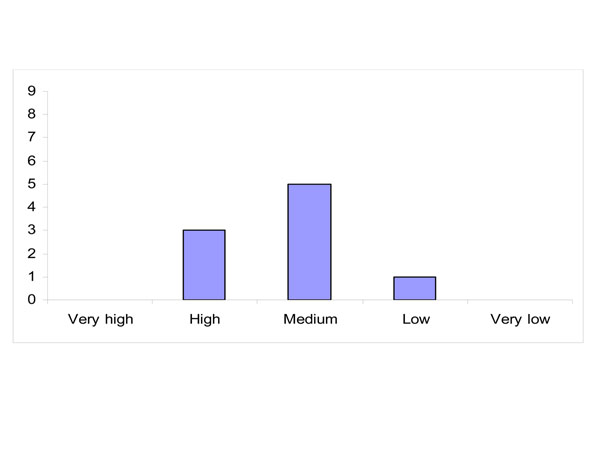
Level of confidence whether policy actions to effectively manage this health risk will become technically (not politically) feasible within the next five years (distribution of answers)

The experts rated their confidence in the possibility that policy actions to effectively manage this health risk will become technically (not politically) feasible within the next five years, confidence was rated lower than their confidence that conducting more scientific research will yield decisive knowledge within the next five years (Figure 4).

Most experts have medium confidence in this respect as while some relevant policy actions are feasible, there is disagreement about their effectiveness. Reasons for high confidence are the availability of both scientific knowledge and of good examples; however concern that the main element that is still missing being political will. Low confidence is attributed due to the fact that current actions do not seem to result in convincing positive effects.

There is consensus among the health experts that there is sufficient evidence to justify policy measures to decrease the existing health risks associated with the stressors based on the current scientific knowledge. Main discussion points are mentioned below:

- Scientific evidence on both health effects and effective solutions is available

- Enough is known for prevention, however criteria for setting priorities policy action may favour reactive measures even though prevention is more effective Exposure to known health stressors will rise, so action is needed; research based evidence required to determine the actions that will be most effective

- Definitive knowledge is not always a prerequisite for policy action

### Follow-up workshop

Amongst the workshop participants there was a concern that the composition of the group of experts, especially the workshop panel, may bias the responses and conclusions. Especially since each person does not consider him/herself to be an expert in all research areas examined by the evaluation. However HENVINET representatives at the workshop observed that “at home” (in their country, institute or department) all participants are expected to have an expert opinion on all parts of the causal diagram. Some of the panel members concurred that they answer these kinds of questions from a general understanding they have based on current scientific knowledge.

There was consensus among the experts that at least no important pathway was missing in the causal diagrams presented. They also agreed that the relevance of different stressors and health risks could be different within different regions in Europe.

The workshop participants had been asked to prioritize the most important pathways. Since the causal diagram was intended for asthma/allergies and respiratory health, many of the experts said this influenced their rating (i.e. some had given heat a lower ranking than they may have without this focus on the respiratory system). Other experts, however, stressed that increased exposure to heat is the effect that is most likely to occur, and that extreme heat is an important cause of mortality in the elderly, particularly in people with COPD and some other diseases.

When discussing high priority mitigation and prevention of health impacts, the workshop participants identified that mitigation and adaptation strategies are sometimes in conflict. For example air conditioning may prevent heat related mortality, but may increase CO_2_ and particle emissions from power plants. On the other hand sometimes adaptation strategies can double as a mitigation strategy. For example measures to increase active transport reduce traffic emissions also result in reduced health effects due to reduced emissions and ozone formation. The experts emphasised that policy making should take such interrelations into account.

## Discussion

The study perspective was European, which may mean that some global effects of climate change were not considered. The participating experts were mainly from the field of environmental and respiratory research, hence the focus was on asthma/allergies and respiratory endpoints. This was in one respect a limitation, but according to the literature the effects of heat waves, ozone and particles, pollen, flooded buildings etcetera, are strongest on respiratory morbidity and mortality, at least in relative terms. This means that experts in this field can be expected to contribute to the discussions on effects of climate change.

There could possibly have been a selection bias from the online evaluation to the follow-up workshop. During the workshop discussion it seemed that, in general, prioritizing causal elements in the case of climate change induced health risks was not easy. One of the reasons for this was that several of these elements are interrelated, and moreover characterized by huge complexity. Another reason was that experts felt somewhat biased by their own expertise, and were sometimes tempted to attribute higher priority to issues within their own expertise or research interest. However, after the follow-up workshop the answers from the first questionnaire were studied by both the workshop panel and the rest of the respondents, showing no striking differences in the answers between these two groups.

Restricting the focus on how respiratory diseases are expected to be affected by climate change may have led some experts to place less emphasis on the effects of heat waves than they may have otherwise done. Among the experts that later participated at the follow-up workshop however, the highest ranking was given to extreme heat (2.89), this is in agreement with research and the IPCC who predict with high certainty, an increase in heat waves.

During the workshop discussions it became clear that all participating experts found the current scientific evidence on health effects from climate change sufficient to take policy actions, even though there still are a lot of unknowns. Despite a high confidence rating that decisive new knowledge will be produced within the next five years, there was much less confidence that decisive policy actions will become possible within the same time frame.

## Conclusions

A group of experts in environmental and respiratory medicine identify clear health risks associated with climate change. Direct health effects of more severe heat waves are an obvious threat. Increasing ozone levels are also seen as a likely health problem. While the researchers do not regard themselves as experts in all related topic areas, they concur that they provide opinion in their role as an expert in public health or as a researcher. A common perception is that there is already a basis for action and prevention, but less confidence that the associated adaptation and mitigation policies will have an evidence base within the same timeframe.

## Competing interests

No competing interests are reported.

## Authors' contributions

All authors planned this work. MKvK, MJK, AB, AY, BF and LB designed the causal model questionnaire. BF, LB and AY evaluated the questionnaire results. BF, LB and HK arranged the follow up workshop. BF wrote the manuscript. HK and LB made the first revision. All authors approved the final version.

## Supplementary Material

Additional file 1 **Experts assessing the causal diagram** List of experts that have been assessing the causal diagram on asthma and allergies Click here for file
