# Supplementary material for: An expert assessment on climate change and health – with a European focus on lungs and allergies
Source: Environ Health. 2012 Jun 28;11(Suppl 1):S4. doi: 10.1186/1476-069X-11-S1-S4 (PMC3388443; doi:10.1186/1476-069X-11-S1-S4)
Supplement: Additional file 1 — Experts assessing the causal diagram List of experts that have been assessing the causal diagram on asthma and allergies [file 1476-069X-11-S1-S4-S1.pdf]

Additional file 1 - Experts assessing the causal diagram on asthma and allergies

Andersen, Zorana,
Department of Biostatistics, Copenhagen University, Copenhagen, Denmark,
zorana@cancer.dk

Ayres, Jon
Institute of Occupational and Environmental Medicine, Birmingham University, UK.
j.g.ayres@bham.ac.uk

Brunekreef, Bert
Institute for Risk Assessment Sciences, Utrecht University, Utrecht, the Netherlands
B.Brunekreef@iras.uu.nl

Cecchi, Lorenzo,
Interdepartmental Centre of Bioclimatology , University of Florence, Italy,
lorenzo.cecchi@asf.toscana.it

Forastiere, Francesco
Rome Environmental Health Authority
Department of Epidemiology
forastiere@asplazio.it

Frei, Thomas
Federal Office of Meteorology and Climatology, Zurich, Switzerland.
thomas.frei@meteoswiss.ch

Garcia-Mozo, Herminia
Department of Botany, Ecology and Plant Physiology, Universidad de Cordoba, Cordoba, Spain.
bv2gamoh@uco.es

Heinrich, Joachim,
Helmholtz Zentrum München, German Research Center for Environmental Health, Institute of Epidemiology, Munich, Germany
joachim.heinrich@gsf.de

Janson, Christer
Dept for Medical Sciences, Uppsala University, Uppsala, Sweden (contacted per telephone).
christer.janson@medsci.uu.se

Krämer, Ursula
Institut für Umweltmedizinische Forschung (IUF) at the Heinrich-Heine-University of Düsseldorf, Auf'm Hennekamp50, 40225
Düsseldorf, Germany
kraemeru@uni-duesseldorf.de

Melén, Erik
Institute of Environmental Medicine, Karolinska Institutet, Stockholm, Sweden.
erik.melen@ki.se

Moshammer, Hans,
Institute of Environmental Health, Medical University of Vienna, Vienna, Austria
hanns.moshammer@meduniwien.ac.at

Norbäck, Dan,
Department of Medical Sciences, Occupational and Environmental Medicine, Uppsala University
dan.norback@medsci.uu.se

Priftis, Kostas
Department of Allergy-Pneumonology Penteli Children's Hospital P. Penteli - Athens, Greece,
kpriftis@otenet.gr

Schlink, Uwe
The Department of Human Exposure Research and Epidemiology, Heimholtz-Zentrum für Umweltforschung GmbH–UFZ, Leipzig-Halle; Germany
schlink@expo.ufz.de

Viegi, Giovanni
Pulmonary Environmental Epidemiology Unit, CNR Institute of Clinical Physiology, Pisa, Italy.
viegig@ifc.cnr.it

		Responded to questionnaire		Participated in workshop
		I	II	
Zorana Andersen	Denmark	1	1	1
Isabella Annesi-Maesano	France	1		
Jon Ayres	Great Britain	1		
Bert Brunekreef	Netherlands	1		
Lorenzo Cecchi	Italy	1	1	1
Francesco Forastiere	Italy	1	1	1
Thomas Frei	Germany	1		
Herminia Garzia-Mozo	Spain	1		
Joachim Heinrich	Germany	1	1	1
Christer Janson	Sweden	1	1	
Erik Melén	Sweden	1		
Hanss Moshammer	Austria	1	1	1
Dan Norbäck	Sweden	1	1	1
Kostas N Priftis	Greece	1	1	1
Uwe Schlink	Germany	1		
Giovanni Viegi	Italy			
